# Association between Serum Uric Acid to HDL-Cholesterol Ratio and Nonalcoholic Fatty Liver Disease in Lean Chinese Adults

**DOI:** 10.1155/2020/5953461

**Published:** 2020-03-23

**Authors:** Ya-Nan Zhang, Qin-Qiu Wang, Yi-Shu Chen, Chao Shen, Cheng-Fu Xu

**Affiliations:** ^1^Department of Geriatrics, Zhejiang Provincial People's Hospital, Hangzhou, China; ^2^Department of Gastroenterology, The First Affiliated Hospital, Zhejiang University School of Medicine, Hangzhou 310003, China; ^3^Health Management Center, The First Affiliated Hospital, Zhejiang University School of Medicine, Hangzhou 310003, China

## Abstract

**Methods:**

A cross-sectional study was performed among 6285 lean Chinese adults (body mass index < 24 kg/m^2^) who took their annual health checkups. NAFLD was diagnosed based on hepatic ultrasound examination, with exclusion of other etiologies.

**Results:**

Of 6285 lean participants enrolled, 654 NAFLD cases were diagnosed. The overall NAFLD prevalence was 10.41%, and the prevalence was 15.45% and 7.16% in men and women, respectively. UHR was significantly higher in NAFLD patients than in controls (14.25 ± 5.33% versus 10.09 ± 4.23%, *P* < 0.001). UHR quintiles were positively associated with NAFLD prevalence, which was 1.91% in the first UHR quintile and increased to 3.58%, 7.81%, 14.17%, and 24.54% in the second, third, fourth, and fifth quintile groups, respectively (*P* < 0.001 for trend). Multivariate logistic regression analysis showed that UHR was independently associated with an increased risk of NAFLD (odds ratio: 1.105; 95% CI: 1.076–1.134; *P* < 0.001). Sensitivity analysis showed that UHR remained significantly associated with NAFLD in lean participants with normal range of serum uric acid and HDL-cholesterol levels.

**Conclusions:**

UHR was significantly associated with NAFLD and may serve as a novel and reliable marker for NAFLD in lean adults.

## 1. Introduction

Nonalcoholic fatty liver disease (NAFLD) is a common chronic liver disease worldwide, with a prevalence of 25% in general adults [1–3]. The spectrum of NAFLD ranges from simple steatosis to nonalcoholic steatohepatitis (NASH) and NASH-related fibrosis or cirrhosis [4]. NAFLD has also been increasingly recognized as an important cause for hepatocellular carcinoma and liver-related mortality [5, 6]. Meanwhile, NAFLD is associated with significantly elevated risks of type 2 diabetes mellitus (T2DM), cardiovascular disease, chronic kidney disease, and extrahepatic malignancies [7–9]. Due to its high prevalence and significant clinical importance, NAFLD has become a global public health issue [10].

Despite the fact that obesity is a major risk factor for the development and progression of NAFLD [11], a growing body of evidence showed that NAFLD is also common in nonobese population [12–14]. We previously reported that the prevalence of NAFLD was 7.3% among a nonobese Chinese population, and 8.9% of nonobese individuals developed NAFLD during a 5-year follow-up [15]. A recent meta-analysis that included 55,936 lean/nonobese participants from 45 studies found that the pooled NAFLD prevalence was 15.7% in nonobese population [16]. The authors also reported that NAFLD prevalence in lean/nonobese population showed a general upward trend during recent years [16]. Although nonobese NAFLD patients had lower body mass index (BMI), they showed similar prevalence of NASH and fibrosis to obese NAFLD patients [17]. Prospective studies also showed that nonobese NAFLD patients had significantly increased risks of incident T2DM and cardiovascular disease [18, 19]. Therefore, more attention should be paid to the prevention and management of NAFLD in nonobese population.

The risk factors for NAFLD in nonobese population have not been fully clarified. We previously reported hyperuricemia as an independent risk for NAFLD [20, 21]. Serum uric acid level was reported to be positively correlated with histological severity of NAFLD as well [22]. HDL-cholesterol is an important component of the metabolic syndrome, and NAFLD patients usually have low HDL-cholesterolemia [23]. Recently, uric acid to HDL-cholesterol ratio (UHR) has been reported to be an independent indicator for the metabolic syndrome and diabetic control in patients with T2DM [24, 25]. However, whether UHR is associated with NAFLD, especially lean NAFLD, remains unclear.

In this study, we performed a cross-sectional study to explore the association of UHR with NAFLD in lean Chinese adults. We also investigated whether UHR is associated with risk of lean NAFLD in participants with normal range of serum uric acid and HDL-cholesterol levels.

## 2. Methods

### 2.1. Study Population

The participants enrolled in this study were adults who took their annual health checkups at the First Affiliated Hospital, Zhejiang University School of Medicine during the year of 2019. We excluded certain participants as follows: (i) those with incomplete data on anthropometric or biochemical parameters or hepatic ultrasound results; (ii) those who were overweight or obese with BMI over 24 kg/m^2^; (iii) those with daily alcohol intake higher than 30 g for men or 20 g for women; and (iv) those with a self-reported history of viral, autoimmune, or other forms of chronic liver disease. A total of 6285 participants were enrolled in the final analysis.

The study protocol was approved by the Ethical Committee of the First Affiliated Hospital, Zhejiang University School of Medicine. Written consent was not required because of the retrospective observational design of the study. The participant information was anonymized prior to analysis.

### 2.2. Anthropometric Measurements

Anthropometric parameters were measured as we previously described [26, 27]. All the participants were asked to fast overnight and take the anthropometric measurements on the next morning. Standing height and body weight were measured with light clothing without shoes, and BMI (kg/m^2^) was calculated as the body weight (kg) divided by the standing height (m) squared. Systolic and diastolic blood pressures at rest were measured using an analog sphygmomanometer. Waist circumference was measured in the standing position at the level of the umbilicus using a flexible anthropometric tape.

### 2.3. Biochemical Measurements

Overnight fasted blood samples were obtained for the analysis of biochemical variables including serum liver enzymes, triglycerides, total cholesterol, HDL-cholesterol, uric acid, and glucose. The variables were measured without freezing, using a Hitachi 7600 autoanalyzer (Hitachi, Tokyo, Japan) with standard methods.

### 2.4. Hepatic Ultrasound Examinations

Hepatic ultrasound examinations were performed to screen fatty liver. The examinations were conducted by experienced ultrasonographists using an ACUSON Sequoia 512 ultrasound machine with a 3.5 MHz probe (Siemens, Mountain View, CA). The ultrasonographists were not aware of the study design and the clinical information. The ultrasonic diagnosis of fatty liver was based on well-established criteria suggested by the Chinese Liver Disease Association [28]. NAFLD was diagnosed based on the hepatic ultrasound examination after the exclusion of alcoholic, viral, autoimmune, or other forms of chronic liver disease.

### 2.5. Statistical Analysis

The statistical analyses were conducted using SPSS 18.0 software for Windows (SPSS Inc., Chicago, IL). Quantitative variables were reported as mean ± standard deviation or as median and interquartile range, as appropriate. Student's *t*-tests, Mann–Whitney *U* tests, or chi-square tests were applied for comparisons of the variables. Univariate and multivariate stepwise logistic regression analysis (backward: Wald; cutoff for entry: 0.05, for removal: 0.10) was performed to identify factors associated with risk of NAFLD. A two-sided *P* value of less than 0.05 was considered to be statistically significant.

## 3. Results

### 3.1. Clinical Characteristics of the Study Population

In this study, a total of 6285 lean participants with a mean ± standard deviation age of 45.17 ± 12.70 years were enrolled, among which 654 were diagnosed as NAFLD. The overall NAFLD prevalence was 10.41%, and the prevalence was 15.45% and 7.16% in men and women, respectively. Clinical characteristics of the study participants with or without NAFLD are compared in Table 1. NAFLD patients had higher male/female ratio, older age, higher BMI, bigger waist circumference, and higher systolic and diastolic blood pressure than controls. NAFLD participants also had higher serum levels of *γ*-glutamyl transpeptidase, triglyceride, total cholesterol, glucose, and uric acid, but lower HDL-cholesterol levels than controls (Table 1). A noticeable finding is that UHR was significantly higher in NAFLD patients than that in controls (14.25 ± 5.33% versus 10.09 ± 4.23%, *P* < 0.001).

### 3.2. Association between UHR Quintiles and Prevalence of NAFLD

To further explore the association between UHR and NAFLD, we classified all the participants into quintiles according to their UHR values as follows: quintile 1: UHR < 6.77%; quintile 2: 6.77% ≤ UHR < 8.54%; quintile 3: 8.54% ≤ UHR < 10.70%; quintile 4: 10.70% ≤ UHR < 13.73%; and quintile 5: UHR ≥ 13.73%. We found a positive correlation between UHR quintiles and the NAFLD prevalence, which was 1.91% in the first UHR quintile and increased to 3.58%, 7.81%, 14.17%, and 24.54% in the second, third, fourth, and fifth quintile groups, respectively (Table 2). This finding suggested that participants with higher UHR are more likely to have NAFLD than those with lower UHR.

### 3.3. Association between UHR and Risk of NAFLD

Logistic regression analyses were performed to identify risk factors of NAFLD. We observed that UHR was significantly associated with an increased risk of NAFLD with an odds ratio (95% CI) of 1.176 (1.157–1.195) in the univariate model (Table 3). After being adjusted for age, gender, BMI, and other variables that are associated with risk of NAFLD in Table 3, UHR remained significantly associated with increased odds of NAFLD (odds ratio: 1.105; 95% CI: 1.076–1.134; Table 4). This finding further supported a significant positive association between UHR and NAFLD.

### 3.4. Sensitivity Analysis

We excluded participants with hyperuricemia (serum uric acid ≥ 7 mg/dl) and those with low HDL-cholesterolemia (serum HDL-cholesterol ≤ 40 mg/dl) and performed sensitivity analysis. Of 4843 participants with normal range of serum uric acid and HDL-cholesterol levels, 328 had NAFLD. We found that NAFLD patients also had higher UHR than controls (10.78 ± 2.55% versus 8.64 ± 2.69%, *P* < 0.001). Multivariate logistic regression analysis adjusted for all variables in Table 3 showed that UHR remained independently associated with risk of NAFLD with an odds ratio (95% CI) of 1.179 (1.113–1.248). These findings indicated that, even though within normal range of serum uric acid and HDL-cholesterol levels, UHR remains significantly associated with NAFLD in lean Chinese adults.

## 4. Discussion

In this study, we provided evidence that UHR was positively associated with NAFLD in a lean Chinese population. First, UHR was significantly higher in NAFLD patients than in controls. Second, UHR quintiles were positively associated with prevalence of NAFLD. Third, UHR was independently associated with an increased risk of NAFLD both in univariate and multivariate regression models. Fourth, the sensitivity analysis showed that UHR remained significantly associated with NAFLD in lean participants with normal range of serum uric acid and HDL-cholesterol levels. These findings suggested that UHR might serve as a novel and reliable marker for lean NAFLD.

NAFLD is a major liver disease that is closely associated with obesity. Recent studies reported that the NAFLD prevalence is as high as around 15% in nonobese population [16]. It is empirically considered that nonobese NAFLD may have less severe liver damage than obese NAFLD. Unfortunately, similar to obese NAFLD, nonobese NAFLD is also subjected to significantly elevated risks of unfavorable hepatic and metabolic outcomes. For example, lean NAFLD patients have comparably high prevalence of NASH as overweight patients do (40.7% versus 45.2%, *P*=0.71) [29], and more than 10% of nonobese NAFLD patients have advanced fibrosis [30]. Given that both NASH and advanced fibrosis in lean patients are related to an increased risk of liver-related mortality [31], prevention, and management of NAFLD in nonobese population is of great clinical significance.

Identification of the risk factors for NAFLD in nonobese population is of great help for screening high-risk individuals. On the one hand, body weight gain within normal range is associated with a significantly increased risk of NAFLD in nonobese population [32]. On the other hand, although with relatively low body weight, nearly 20% of nonobese individuals are metabolically unhealthy and have unfavorable metabolic profiles [33]. Metabolically unhealthy conditions are associated with increased risk of metabolic diseases, which may partially explain why NAFLD is also common in nonobese population [34].

Uric acid is the end product of purine metabolism, and elevated serum uric acid levels significantly increase the risk of NAFLD [35]. We previously identified serum uric acid as an independent risk factor for incident NAFLD in nonobese population [15]. Low HDL-cholesterol is also found to be related to a worse metabolic status, which significantly increases the risk of NAFLD [36]. However, whether normal range of serum uric acid and HDL-cholesterol levels also predict risk of NAFLD in nonobese population has not been investigated previously. Nor is it clear whether the combination of uric acid and HDL-cholesterol predicts the risk of lean NAFLD. In this study, we provided evidence for the first time that UHR was closely associated with NAFLD in a lean Chinese population, and the association remained significant in lean individuals with normal serum uric acid and HDL-cholesterol levels. Our results suggested that UHR might be a novel marker for screening NAFLD in lean population.

The strength of this study lies in its large sample size and its focus on lean population. However, several limitations are acknowledged. First, fatty liver was diagnosed by ultrasound, which may be not sensitive enough to detect mild steatosis. Second, whether UHR is associated with histological severity of NAFLD remains unclear. We observed that UHR was positively correlated with serum liver enzymes (data not shown), indicating that UHR might be associated with the severity of NAFLD. Third, our cross-sectional study could not determine the prospective predictive value of UHR for the development of NAFLD. Further studies are needed to address these issues.

In conclusion, our study showed that UHR is positively associated with NAFLD, and UHR may serve as a novel and reliable marker for NAFLD in lean Chinese adults.

## Figures and Tables

**Table 1:** Clinical characteristics of the study population according to NAFLD categories.

Variables	Controls (*n* = 5631)	NAFLD (*n* = 654)	*t* value	*P* value
Gender (male/female)	2080/3551	380/274	110.195^a^	<0.001
Age (year)	44.53 (12.73)	50.73 (10.97)	11.964	<0.001
Body mass index (kg/m^2^)	21.19 (1.80)	22.71 (1.05)	21.163	<0.001
Waist circumference (cm)	76.18 (6.79)	82.93 (5.77)	22.991	<0.001
Systolic blood pressure (mmHg)	117.17 (16.75)	127.56 (16.76)	15.021	<0.001
Diastolic blood pressure (mmHg)	71.06 (10.78)	78.13 (10.52)	15.916	<0.001
*γ*-Glutamyl transpeptidase (U/L)	14.00 (11.00–21.00	26.00 (17.00–39.00)	20.927^b^	<0.001
Triglyceride (mg/dl)	85.00 (62.87–117.76)	148.76 (106.25–203.21)	24.390^b^	<0.001
Total cholesterol (mg/dl)	174.04 (32.88)	184.07 (36.24)	7.301	<0.001
HDL-cholesterol (mg/dl)	52.99 (13.10)	43.41 (10.36)	18.045	<0.001
Fasting blood glucose (mg/dl)	87.22 (12.95)	97.03 (23.42)	16.494	<0.001
Serum uric acid (mg/dl)	4.93 (1.21)	5.80 (1.34)	17.088	<0.001
UHR (%)	10.08 (4.22)	14.25 (5.33)	23.226	<0.001

Data are expressed as mean (standard deviation) or median (interquartile range). ^a^*χ*^2^ value; ^b^*Z* value; HDL, high-density lipoprotein; NAFLD, nonalcoholic fatty liver disease; UHR, uric acid to HDL-cholesterol ratio.

**Table 2:** Association between UHR quintiles and prevalence of NAFLD in lean participants.

UHR quintiles	Total	NAFLD	PR%	PR	*χ* ^2^	*P* value
Quintile 1	1257	24	1.91	1.00		
Quintile 2	1256	45	3.58	1.88		
Quintile 3	1254	98	7.82	4.09		
Quintile 4	1263	179	14.17	7.42		
Quintile 5	1255	308	24.54	12.85	457.297	<0.001

PR%, prevalence rate; PR, prevalence ratio; NAFLD, nonalcoholic fatty liver disease; UHR, uric acid to HDL-cholesterol ratio.

**Table 3:** Univariate analysis for factors associated with risk of NAFLD.

Variables	*β*	SE	Wald *χ*^2^	OR (95% CI)	*P* value
Male gender	0.862	0.084	105.469	2.368 (2.009–2.791)	<0.001
Age (year)	0.037	0.003	135.145	1.037 (1.031–1.044)	<0.001
Body mass index (kg/m^2^)	0.716	0.038	361.419	2.047 (1.901–2.203)	<0.001
Waist circumference (cm)	0.166	0.008	415.905	1.181 (1.162–1.200)	<0.001
Systolic blood pressure (mmHg)	0.032	0.002	202.452	1.032 (1.028–1.037)	<0.001
Diastolic blood pressure (mmHg)	0.056	0.004	227.420	1.057 (1.050–1.065)	<0.001
*γ*-Glutamyl transpeptidase (U/L)	0.016	0.002	116.985	1.017 (1.014–1.020)	<0.001
Triglyceride (mg/dl)	0.010	0.001	355.989	1.010 (1.009–1.011)	<0.001
Total cholesterol (mg/dl)	0.009	0.001	52.154	1.009 (1.006–1.011)	<0.001
Fasting blood glucose (mg/dl)	0.031	0.002	149.196	1.031 (1.026–1.036)	<0.001
UHR (%)	0.162	0.008	388.280	1.176 (1.157–1.195)	<0.001

*β*, partial regression coefficient; NAFLD, nonalcoholic fatty liver disease; OR, odds ratio; CI, confidence interval; SE, standard error of partial regression coefficient; UHR, uric acid to HDL-cholesterol ratio.

**Table 4:** Multivariate analysis for factors associated with risk of NAFLD.

Variables	*β*	SE	Wald *χ*^2^	OR (95% CI)	*P* value
Male gender	0.630	0.131	23.319	1.878 (1.454–2.426)	<0.001
Age (year)	0.011	0.005	5.004	1.011 (1.001–1.020)	0.025
Body mass index (kg/m^2^)	0.385	0.051	57.291	1.024 (1.014–1.033)	<0.001
Waist circumference (cm)	0.071	0.012	34.368	1.074 (1.048–1.099)	<0.001
Diastolic blood pressure (mmHg)	0.023	0.005	24.286	1.024 (1.014–1.033)	<0.001
*γ*-Glutamyl transpeptidase (U/L)	0.005	0.001	12.268	1.005 (1.002–1.008)	<0.001
Triglyceride (mg/dl)	0.004	0.001	26.362	1.004 (1.000–1.006)	<0.001
Fasting blood glucose (mg/dl)	0.015	0.003	33.819	1.015 (1.010–1.020)	<0.001
UHR (%)	0.100	0.013	56.269	1.105 (1.076–1.134)	<0.001

*β*, partial regression coefficient; NAFLD, nonalcoholic fatty liver disease; OR, odds ratio; CI, confidence interval; SE, standard error of partial regression coefficient; UHR, uric acid to HDL-cholesterol ratio.

## Data Availability

The data used to support the findings of this study are available from the corresponding author upon request.
